# Generativity across adulthood: how nature exposure and future time perspective shape motivation for social and ecological engagement

**DOI:** 10.1080/00049530.2024.2428306

**Published:** 2024-11-18

**Authors:** Selma Korlat, Christina Ristl, Jana Nikitin

**Affiliations:** Department of Developmental and Educational Psychology, Faculty of Psychology, University of Vienna, Vienna, Austria

**Keywords:** Social generativity, ecological generativity, nature exposure, future time perspective, adulthood

## Abstract

**Objective:**

The motivation to leave a legacy for future generations and society’s continuity is an important aspect of adult development. However, the shorter time horizon that comes with ageing might lead to prioritising more immediately rewarding goals than long-term society-beneficial (e.g., climate-proactive) goals. This study investigates the role of nature exposure and future time perspective, as well as their joint interplay in the context of social and ecological generativity across adulthood.

**Method:**

In total, 115 individuals aged 18–85 years (*M* = 38.10 years, *SD* = 16.53 years, age-range 18-85; 67% female) participated in an experience sampling study and reported their future time perspective and social and ecological generativity at the baseline, and nature exposure in their daily situations.

**Results:**

The results of the analyses using aggregated data set showed that nature exposure facilitates the concern for future generations and the environment in middle-aged and older (but not in younger) adults with shorter future time perspective.

**Conclusion:**

Nature exposure can be used to enhance motivation for societal involvement in middle and older adulthood, which in turn could contribute to the well-being and sustainability of future generations.

## KEYPOINTS

**What is already known about this topic:**

1. The motivation to leave a legacy for future generations (generativity) is an important aspect of adult development, with social (care for people) and ecological (care for environment) generativity being prominent not only in midlife but in early and late adulthood as well.
2. Generative feelings towards the environment seem to emerge from direct experiences with nature.
3. Older adults typically have a shorter future time perspective, leading them to prioritise more immediately rewarding goals than long-term goals.

**What this topic adds:**

1. Longer future time perspective positively predicts social and ecological generativity.
2. Nature exposure facilitates the concern for future generations and the environment in older (but not in younger) people with shorter future time perspective.
3. As such, nature exposure could serve as a potential intervention tool for boosting motivation in older adults with shrinking time horizons to protect future generations and the environment, enhancing their unique contributions to the well-being of future generations.

Generativity, a concept first articulated by Erikson (1963), describes the innate human motivation to nurture and support future generations. Initially conceptualised as a stage primarily experienced during midlife, recent research has expanded the understanding of generativity as a significant developmental aspect throughout the entire adult lifespan (Alisat et al., 2014; McAdams, 2001). Nowadays, society’s continuation and environmental issues and are among the most urgent and important concerns (e.g., Heeren & Asmundson, 2023; Morrison et al., 2023). Addressing these challenges requires action across all age groups, fostering prosocial attitudes for the well-being of next generations and the Earth’s future (Di Fabio & Svicher, 2023; Schoklitsch & Baumann, 2011). This manuscript examines generativity encompassing social and ecological aspects, focusing on how factors such as nature exposure and future time perspective shape generativity, seeking to understand their interactive effects on adults’ motivation to contribute positively to the community and their environment.

## Generativity across adulthood

The motivation to nurture future generations and preserve humankind and its resources is an important developmental aspect in adulthood (Alisat et al., 2014; Erikson, 1963). Although Erikson initially described generativity as a main stage in midlife, leaving a legacy that will “outlive the self” (Kotre, 1984, p. 10) can be a critical driving force for engagement in societally beneficial activities in the entire adult course of life (McAdams, 2001). For example, care for the next generations and society as a whole remains predominant until late adulthood (Schoklitsch & Baumann, 2011; Vaillant, 2002). The so-called *grand-generativity* (Erikson, 1986) recognises that older adults can continue to show a strong commitment to relevant societal issues (Villar et al., 2023). Empirical evidence supports this notion, showing that generativity might play a key role in older people’s engagement in community-oriented activities (e.g., Pinazo-Hernandis et al., 2023; Villar, 2012; Villar & Serrat, 2021). On the other hand, generative concern has been found to emerge already in young adulthood (Lawford et al., 2013; Pratt et al., 2009). The studies on *early generativity* show that the results on generative concern in the domain of community and environmentalism are comparable between emerging adults and middle-aged adults (for a review see Pratt & Lawford, 2014). Thus, empirical evidence confirms McAdams (2001) notion that generativity does not wait until midlife to unfold, nor does it lose its function with the beginning of old age. Consequently, the construct of generativity is redefined and broadened not only to other stages of the life course but to new forms of generativity as well (e.g., Di Fabio & Svicher, 2023; Kim et al., 2017). Specifically, *social* and *ecological generativity* arose as equally prominent across the entire adulthood, as opposed to biological and parental (see Kotre, 1984) which are more dominantly related to typical midlife generativity.

## Social and ecological generativity and nature exposure

Social generativity refers to caring for other (younger) people and “generally contributing to the strength and continuity of subsequent generations” (Snarey, 1993, p. 22 in Schoklitsch & Baumann, 2011). Ecological generativity refers to preserving the environment for future generations (Schoklitsch & Baumann, 2011). In the present study, generative concerns related to people (social generativity) and the environment (ecological generativity) are regarded through a unified framework as fundamental generative pillars related to future societal and environmental matters. Both have been found to predict engagement in pro-environmental and community-related activities, in both younger and older adults (Alisat et al., 2014; Pinazo-Hernandis et al., 2023; Pratt et al., 2013). For instance, in a study with young and middle-aged adults, Zaval et al. (2015) found a positive correlation between legacy motivation and pro-environmental behaviours and intentions. Moreover, in a subsequent experiment, they demonstrated that priming legacy motives increased climate-change beliefs, pro-environmental intentions, and donations to an environmental charity (Zaval et al., 2015).

Generativity has also been found to be a key mediator between a close personal connection with nature and environmental engagement (Alisat et al., 2014). These authors argue that the generative feelings towards the environment seem to emerge from direct experiences with nature (Alisat et al., 2014). Thus, nature exposure is a potential facilitating factor for generativity. Empirical evidence shows that meaningful interaction with nature can foster a sense of purpose and responsibility towards the environment and society, which can drive individuals to engage in actions that benefit others and the planet (for a review see Pritchard et al., 2020). Considerable evidence from observational and intervention studies indicates that time spent in nature leads to increased perceived value for connectedness to nature and greater pro-environmental attitudes and behaviours (see DeVille et al., 2021; Martin et al., 2020). Moreover, connecting with nature can increase empathy and compassion (Fido & Richardson, 2019) – traits that are crucial for generativity as they encourage individuals to care for future generations and community goods.

## The role of future time perspective

Another factor that is closely connected to social and ecological generativity is future time perspective (DiFabio & Svicher, 2023), as concern for continuity and perception of the future are interconnected (Morselli, 2013) and time is a pivotal resource in that context (Pahl et al., 2014). Future time perspective refers to human perception of their future through the prism of the remaining lifetime (Carstensen et al., 1999). Socioemotional Selectivity Theory (SST; Carstensen et al., 1999) posits that young adults typically have open-ended time horizons, enabling them to invest time and resources in activities with long-term results. Older adults, on the other side, typically perceive their time as limited, leading them to prioritise emotionally meaningful activities and relationships that provide immediate satisfaction and fulfilment (Freund, 2024).

Environmental psychology has shown future time perspective to positively predict pro-environmental attitudes and environmental preservation (for a review see Milfont et al., 2012; Tucholska et al., 2024), depicting the long-term future time perspective as a precondition for concern about society’s and Earth’s future beyond the duration of one’s own lifetime (Morselli, 2013). In light of this rationale, a longer future time perspective in younger age groups should facilitate social and ecological generativity, whereas a shorter time perspective among older adults could play a hindering role. Some developmental studies confirmed this notion, arguing that although older adults or those with a limited time perspective may have strong generative desires, they might be less likely to include these goals in their “bucket list” when time is very restricted (e.g., Chu et al., 2018; Hösch et al., 2023). On the other hand, other developmental studies showed that as time horizons shrink, emotionally meaningful goals (such as generativity) become more important (Lang & Carstensen, 2002) and people shift towards self-transcendent goals that do not entail future personal benefits (Brandtstädter et al., 2010).

## The present study

Engagement in relevant societal issues evokes both social (individual vs. collective interests) and temporal aspects (short- vs. long-term interests, i.e., immediate vs. delayed consequences of individual’s actions; Tucholska et al., 2024). As human beings have a limited amount of time resources that can be used to preserve human and natural resources, psychological variables that help to explain decision-making process in the context of future societal and environmental matters, should be observed developmentally through the prism of time perspective and finitude of life. As nature exposure can have a pivotal facilitating role in the matter (Alisat et al., 2014), the aim of this study is to investigate the interplay between nature exposure and future time perspective in the context of social and ecological generativity. Despite both being recognised as important factors for generativity, no previous studies, to our knowledge, investigated their joint effects on generative concerns related to future generations and environment. Moreover, it seems to still be an open question whether longer or shorter time perspective fosters generativity. The current study aims to test: 1) Are nature exposure and future time perspective associated with generativity, both independently and in interaction? and – since age and future perspective are related but not equal (Liao & Carstensen, 2018) − 2) Does the interaction between nature exposure and future time perspective differ between younger and older people? Understanding age differences in this context is essential to understand the legacy motivation in different adulthood stages that can be used to foster engagement in societal issues, such as climate change action. As previous evidence suggested that education level (McAdams et al., 1992), as an indicator of socioeconomic status, could affect generativity, we controlled for it in the analyses.

## Method

### Participants

Participants were part of the “Quality of Daily Life” experience sampling study (Nikitin et al., 2022, Study 2). The convenience sample was recruited from Vienna and surrounding areas. Out of 159 initial participants, 44 individuals were excluded from analyses due to missing values on nature exposure measures. The final sample of 115 individuals was aged 18–85 years (*M* = 38.10 years, *SD* = 16.53 years, age-range 18–85; 67% female). Among participants, 66.1% were married or in a relationship, 57.9% reported having a university degree and 60.9% reported being employed.

### Procedure

After completing a baseline questionnaire (online), daily assessments were conducted with the open-source experience sampling app ESM-Quest (Goetz et al., 2024). The baseline questionnaire contained several scales related to life experiences, such as emotion regulation, avoidance and approach motivation, gains and losses, as well as generativity items, future time perspective scale, and demographic information (e.g., age, gender, family status and education). For seven consecutive days, participants received six randomised signals in 2-hr intervals (between 8 am and 8 pm) and were instructed to describe their current situation in terms of activity and surroundings. The 115 participants completed a total of 2,906 entries (1,574 in the group of younger adults and 1,332 in the group of middle and older adults).

### Measures

#### Social and ecological generativity

A 12-item questionnaire (adapted from Schoklitsch & Baumann, 2011) that contains items on social and ecological generativity as a single scale was used (sample items: “I am interested in the future development of mankind”, “I bring about positive changes in society or my environment”, “I am politically, socially, or environmentally active”; *α* = .96, *M* = 4.24, *SD* = 1.40). Items were answered on a scale from 1 = “does not apply at all” to 5 = “fully applies”.

#### Future time perspective

Perception of subjective remaining lifetime and opportunities in the future were captured using 10 items (Lang & Carstensen, 2002; sample items“: “Many opportunities await me in the future”, “Most of my life still lies ahead of me”, “I have the sense that time is running out”, *α* = .79, *M* = 4.37, *SD* = 1.12). Reversed items were recoded so that higher value indicates a longer future time perspective on a scale from 1 = “does not apply at all” to 7 = “fully applies”.

All items used to measure social and ecological generativity, as well as future times perspective can be found in the supplementary material.

#### Daily nature exposure

Subjectively perceived closeness to nature in everyday situations was measured with the item: “How close to nature was the environment?” wherein participants had to indicate whether during the momentary assessment they were 1 = “not at all close to nature”, 2 = “somewhat close to nature”, 3 = “quite close to nature”, 4 = “in pure nature” (*M* = 1.71, *SD* = 0.51).

### Analyses

To investigate the relationship between the variables under the study, we used linear multiple regression analysis calculated using SPSS v29. Although nature exposure in everyday situations is nested within individual participants (as level-1 predictor), future time perspective and generativity were measured at the baseline (as level-2 moderator and outcome variable). In the lack of level-1 outcome variables, measurement time points of nature exposure were aggregated for each participant. We calculated a hierarchical regression analysis with all predictors (nature exposure, future time perspective and age-group) as main effects in Model 1, their two-way interactions in Model 2, and the three-way interaction in Model 3. The generativity scale was the outcome variable. The coefficient of determination R^2^ was used to assess the amount of explained variance. Nature exposure and future time perspective were grand-mean centred. Education level was entered as a control variable in all models.

## Results

Correlations between the variables are presented in Table 1. Results showed a significant positive correlation between age and nature exposure, as well as between age and generativity, and a significant negative correlation between age and future time perspective. All other relationships between the variables were not significant, all *ps* > .05.

**Table 1.** Bivariate correlations of the study variables.

Variable	1	2	3	4
1. Nature exposure	1			
2. Future time perspective	−.03	1		
3. Age	.24^*^	−.68^**^	1	
4. Generativity	.18	.07	.19^*^	1

*p < .05.

**p < .001.

### Main effects and interaction effects

Model 1 with predictors investigating main effects was significant, *F*(4,109) = 7.802, *p* < .001, *R*^*2*^ = 22.3%. As reported in Table 2, the results showed a non-significant association between nature exposure and generativity, but a significant positive association between future time perspective and generativity, as well as between age and generativity. Longer future time perspective and older age were positively associated with generativity. Education level was also a significant positive predictor of generativity.

**Table 2.** Multiple regression models for main effects, two-way interactions and three-way interaction between nature exposure, future time perspective and age with social and ecological generativity as outcome variable and education level as control variable.

Variable	b	SE	β	t	p
Model 1					
Intercept	2.258	.615	–	3.672	**<.001**
Nature exposure	.358	.246	.129	1.451	.150
FTP	.428	.137	.**343**	3.125	.**002**
Age	.035	.009	.**414**	3.694	**<.001**
Education level	.462	.140	.**290**	3.305	.**001**
Model 2					
Intercept	2.366	.630	–	3.753	**<.001**
Nature exposure	.381	.252	.138	1.517	.133
FTP	.466	.140	.**373**	3.319	.**001**
Age	.038	.010	.**453**	3.798	**<.001**
Education level	.461	.141	.**289**	3.278	.**001**
Nature exposure x FTP	−.084	.265	−.034	−.315	.753
Nature exposure x Age	.003	.018	.019	.175	.862
FTP x Age	.009	.006	.131	1.464	.146
Model 3					
Intercept	2.533	.627	–	4.037	**<.001**
Nature exposure	.053	.298	.019	.179	.858
FTP	.531	.142	.**425**	3.729	**<.001**
Age	.041	.010	.**484**	4.082	**<.001**
Education level	.431	.139	.**271**	3.091	.**003**
Nature exposure x FTP	−.328	.289	−.134	−1.135	.259
Nature exposure x Age	−.009	.019	−.054	−.478	.634
FTP x Age	.013	.007	.182	1.975	.051
Nature exp x FTP x Age	−.031	.016	**−.228**	−1.988	.**049**

FTP = Future time perspective; b = unstandardized regression coefficient; SE*=*Standard errors of regression coefficients. β = standardized regression coefficient (slope). Significant parameters (*p* < .05) are marked bold. The analysis is based on *n* = 114 participants. Nature exposure, age and FTP were grand-mean centred.

Age, future time perspective and education level remained significant positive predictors of generativity in Model 2, which was also significant *F*(7,106) = 4.770, *p* < .001, *R*^*2*^ = 24%. However, none of the two-way interactions were significant.

Model 3 was also significant, *F*(8, 105) = 4.784, *p* < 0.001, and accounted for *R*^*2*^ = 26.7% of the variance. As presented in Table 2, age, future time perspective and education level were significant positive predictors of generativity. The three-way interaction was also significant. To explore the three-way interaction, we divided the sample into two age groups – with those under 39 years of age being grouped as younger adults (*n* = 68, *M* = 25.9 years, SD = 4.4 years; age-range 18–38, 69% females) and those 40 years of age and older being grouped as middle and older adults (*n* = 47, *M* = 55.8 years, SD = 10.5 years, age-range 40–85; 64% females). The age threshold was selected based on empirical and theoretical insights regarding the trajectories of the variables under study throughout adulthood. Generativity, as a developmental task, peaks around the age of 40 (Nelson et al., 2021) and remains relatively stable afterwards, particularly in the domains of social and ecological generativity (Erikson, 1986; Pinazo-Hernandis et al., 2023). Similarly, a lifespan meta-analysis on age-related differences in prosociality, which is closely linked to concerns for others and the environment, shows a peak in midlife, followed by a relatively stable course in older age (Pollerhoff et al., 2024). Moreover, future time perspective starts to shrink at the beginning of mid-adulthood, and it is more influential (diagnostic) for goals and behaviour in the second half of life compared to the entirety of young adulthood when time horizon seems infinite (Carstensen et al., 1999).

**Figure 1.**
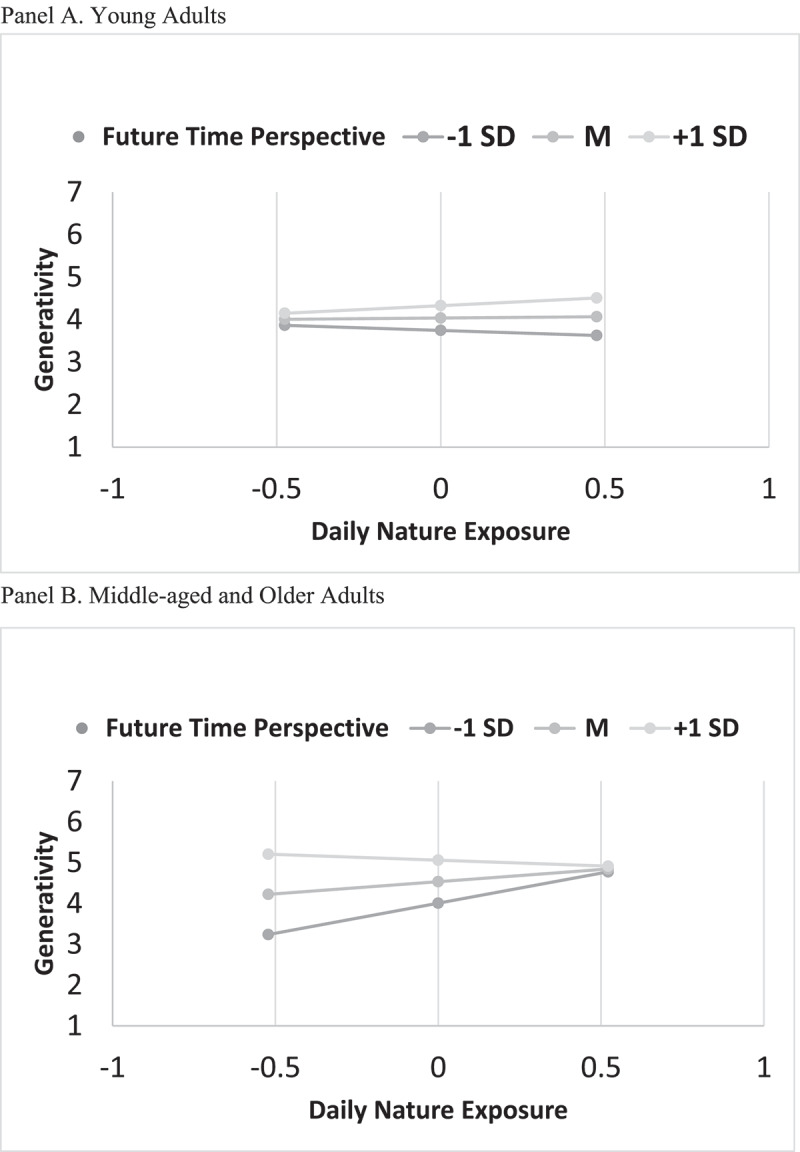
Interplay between nature exposure, future time perspective in social and ecological generativity in young (panel A) and middle-aged and older (panel B) adults.

The interaction between nature exposure and future time perspective in the group of young adults was not significant *F(*3,64) = 1.50, *p* = .224, *R*^*2*^ = 6.56%, whereas in the group of middle and older adults, the interaction effect was significant, *F*(3,43) = 4.93, *p* = .005, *R*^*2*^ = 25.6%. As shown in Figure 1, Panel B, nature exposure was significantly associated with social and ecological generativity in middle/older adults with shorter time perspective (all other slopes were *ps* > .05).

## Discussion

A new paradigm of reframing ageing emphasises the potential of older adults as key players in relevant societal issues, such as climate action, especially because of their developmental generative capacity (Diehl, 2022). Based on that rationale, it is important to better understand factors of social and ecological generativity, especially in adults facing more limited time horizons. While the reframing ageing paradigm focuses primarily on the potential of older adults as key players in societal issues, such as climate action, it is important to also consider the role of middle-aged adults. Middle age represents a life phase where individuals developmentally begin to shift their focus towards generative goals (Nelson et al., 2021) and time horizons star to narrow (Carstensen et al., 1999). Including this age-group in the narrative about social and ecological involvement is equally relevant, as middle-aged adults, too, face shrinking time horizons and can play an influential role in addressing urgent societal issues.

This study highlights the complex interplay between nature exposure, future time perspective, and generativity across adulthood. Our findings demonstrate that future time perspective positively predicts generativity, aligning with previous research suggesting that a longer future outlook fosters concern for long-term societal and environmental issues (Milfont et al., 2012; Morselli, 2013; Tucholska et al., 2024). Notably, middle-aged and older adults with shorter future time perspectives still exhibited high levels of generativity, particularly when exposed to nature. This suggests that nature exposure can act as a powerful facilitator of generativity in middle-aged and older adults when time horizons are perceived as (more) limited.

This is an important finding given that some developmental studies found that older adults or those with shorter time perspectives might be less likely to prioritise generative goals despite their generative desire (e.g., Chu et al., 2018; Hösch et al., 2023). As people age, their perception of time inevitably changes, leading them to focus more on emotionally meaningful goals with immediate rewards (Freund, 2024), which could potentially reduce involvement of adults in long-term generative goals as they are getting older. This could be especially detrimental in the context of relevant societal issues, as many currently pressing societal challenges, such as environmentalism, require action whose impact tends to be delayed and evident only in the distant future. In the context of climate change mitigation efforts, for instance, although older (compared to younger) adults seem to invest efforts in pro-environmental behaviour (Wang et al., 2021; Wiernik et al., 2016), they still exhibit lower levels of concern, collective action and support in climate change initiatives (Ágoston et al., 2024; Poortinga et al., 2019). As generativity has previously been noted as a key organising structure within the environmental domain (Alisat et al., 2014), fostering social and ecological generativity could enhance middle-aged and older adults’ unique contributions in addressing environmental, but also other critical societal challenges, such as globalisation, political polarisation and extremism in order to protect next generations and their future.

As such, nature exposure could serve as a potential intervention tool for boosting motivation in older adults with limited time perception to protect future generations and their well-being. This could consequently promote pro-environmental behaviours (Zaval et al., 2015). Even fifteen years ago, scholars debated about potential factors that could cultivate “long-view” about the future and safeguarding the Earth among older adults (e.g., Moody, 2009), and seems that nature exposure could have such a potential. Moreover, both nature exposure itself (e.g., Jarosz, 2022; Rojas-Rueda et al., 2019) and social activism have numerous positive effects on older adults, including fulfilled purpose and meaning in life (see Diehl, 2022). Similarly, developing sense of legacy and generativity could increase not only community and pro-environmental action, but also well-being and social integration in later life (Gruenewald et al., 2012). Thus, the benefits of nature exposure are manifold, allowing even older adults with shorter time perspectives to position as elders for change and eco-elders, inspired by their generativity and legacy concerns, who can contribute to the continuity and well-being of society and the Earth.

Older age was a constant significant positive predictor of generativity in our study, although previous studies have shown similar levels of generative concern in community- and environment-related issues between emerging adults and middle-aged adults. The generativity questionnaire used (Schoklitsch & Baumann, 2011) includes items on both generative concern and accomplishments, as well as both ecological and social components, but the latter ones may be more prominent. Thus, the finding of tendency to a higher social and ecological generativity score with increasing age in this study is not surprising. Interestingly, although age and future time perspective are negatively correlated, both were positively associated with generativity in Model 1 and Model 2. While future time perspective decreases with age, generativity is positively associated with future time perspective, even if time horizon is restricted. With increasing age from middle adulthood to old age, a progressively narrowing future time perspective may motivate people to focus more on meaningful activities that contribute to others and society. They might feel the need to “give back” and feel motivated to protect future generations before their time runs out. This confirms some notions from previous developmental studies showing that older people do shift to generative goals *despite* and *because* of shrinking time horizons (Brandtstädter et al., 2010; Lang & Carstensen, 2002). However, when the interaction between age, time perspective and nature exposure is added to the model, age is no longer an individual significant predictor of generativity, but their relationship depends on their exposure to the natural environment and their time perspective.

Surprisingly – on the other hand, nature exposure was not found to be a significant predictor of social and ecological generativity. It might be that mere nature exposure is not strong enough to impact generativity and that a more comprehensive measure on nature experience or nature connectedness would yield such an effect.

### Limitations and future directions

Although the current study provides insights into an important interplay between the nature exposure and future time perspective for social and ecological generativity in earlier and later periods of adulthood, some limitations must be considered. First, although the study contains daily assessment data, analyses are conducted with aggregated data points, loosing additional information on daily variance. Future ESM studies should include daily generativity and future time perspective measurements. Second, although the age-range in our study is 18–85 years, most of the sample are young and middle-aged adults. Future studies could include larger samples with broader age-range to investigate differences in social and ecological generativity in all age-groups separately, starting from emerging adulthood or even adolescence to early adulthood, middle-age and different stages in older adulthood. Third, future studies should include more comprehensive measures on nature exposure and nature connectedness, in order to investigate differences in generativity depending on intensity and quality of nature exposure. Similarly, additional variables affecting generativity, also social and ecological dimensions of generativity, could be considered, such as parenthood and family roles, cultural and societal context, or personality traits. Fourth, separate scales for social and ecological generativity could be used in order to disentangle concerns about the future of people and the future of Earth. In addition, measures on community or pro-environmental behaviours and actions could be included to investigate effect beyond generative concern or capacity.

## Conclusion

The results of the current study emphasise the potential of leveraging nature exposure as an intervention to boost generativity among middle-aged and older adults, enhancing their contribution to relevant societal challenges. By highlighting the enduring and adaptable nature of generativity throughout adulthood, this study suggests its critical role in societal issues (e.g., environmental stewardship), offering insights into how individuals in middle and older adulthood can be motivated to leave a positive legacy. These findings might be used for developing strategies to enhance motivation for societal involvement after the age of 40, ultimately contributing to the sustainability and well-being of future generations.

## Supplementary Material

Supplemental Material

## Data Availability

The data that support the findings of this study are available from the corresponding author, SK, upon request.
