# Supplementary material for: Generativity across adulthood: how nature exposure and future time perspective shape motivation for social and ecological engagement
Source: Aust J Psychol. 2024 Nov 18;76(1):2428306. doi: 10.1080/00049530.2024.2428306 (PMC12218478; doi:10.1080/00049530.2024.2428306)
Supplement: Supplemental Material [file RAUP_A_2428306_SM7156.docx]

# Social and Ecological Generativity

In my current life situation…

| Trifft gar nicht zu | |  |  |  |  |  | Trifft genau zu |
| --- | --- | --- | --- | --- | --- | --- | --- |
| 1. … I pass on my experience to other people. | 🞏 | 🞏 | 🞏 | 🞏 | 🞏 | 🞏 | 🞏 |
| 2. … I create works that are an enrichment for other people. | 🞏 | 🞏 | 🞏 | 🞏 | 🞏 | 🞏 | 🞏 |
| 3. … I transmit social, political or cultural values to other people. | 🞏 | 🞏 | 🞏 | 🞏 | 🞏 | 🞏 | 🞏 |
| 4. … I bring about positive changes in society or in my environment. | 🞏 | 🞏 | 🞏 | 🞏 | 🞏 | 🞏 | 🞏 |
| 5. … I am a role model for other people. | 🞏 | 🞏 | 🞏 | 🞏 | 🞏 | 🞏 | 🞏 |
| 6. … I promote other people | 🞏 | 🞏 | 🞏 | 🞏 | 🞏 | 🞏 | 🞏 |
| 7. … I take responsibility for other people. | 🞏 | 🞏 | 🞏 | 🞏 | 🞏 | 🞏 | 🞏 |
| 8. … I help other people to develop themselves. | 🞏 | 🞏 | 🞏 | 🞏 | 🞏 | 🞏 | 🞏 |
| 9. … I pass on knowledge or skills to other people | 🞏 | 🞏 | 🞏 | 🞏 | 🞏 | 🞏 | 🞏 |
| 10. … I care for other people. | 🞏 | 🞏 | 🞏 | 🞏 | 🞏 | 🞏 | 🞏 |
| 11. … I am politically, socially or environmentally active. | 🞏 | 🞏 | 🞏 | 🞏 | 🞏 | 🞏 | 🞏 |
| 12. … I am interested in the further development of mankind. | 🞏 | 🞏 | 🞏 | 🞏 | 🞏 | 🞏 | 🞏 |

# Future Time Perspective

Please assess to what extent these statements about your future apply to you.

|  | Trifft gar nicht zu |  |  |  |  |  | Trifft sehr gut zu |
| --- | --- | --- | --- | --- | --- | --- | --- |
| 1. There are many opportunities waiting for me in the future. | 🞏 | 🞏 | 🞏 | 🞏 | 🞏 | 🞏 | 🞏 |
| 2. I believe that I will have many new goals in the future. | 🞏 | 🞏 | 🞏 | 🞏 | 🞏 | 🞏 | 🞏 |
| 3. My future is full of possibilities. | 🞏 | 🞏 | 🞏 | 🞏 | 🞏 | 🞏 | 🞏 |
| 4. The biggest part of my life lies ahead of me. | 🞏 | 🞏 | 🞏 | 🞏 | 🞏 | 🞏 | 🞏 |
| 5. My future seems infinite to me. | 🞏 | 🞏 | 🞏 | 🞏 | 🞏 | 🞏 | 🞏 |
| 6. In my future life I can still do everything I want to do. | 🞏 | 🞏 | 🞏 | 🞏 | 🞏 | 🞏 | 🞏 |
| 7. In my life I still have a lot of time to make new plans. | 🞏 | 🞏 | 🞏 | 🞏 | 🞏 | 🞏 | 🞏 |
| 8. I have the feeling that my time is running out. | 🞏 | 🞏 | 🞏 | 🞏 | 🞏 | 🞏 | 🞏 |
| 9. My possibilities in the future are limited. | 🞏 | 🞏 | 🞏 | 🞏 | 🞏 | 🞏 | 🞏 |
| 10. With increasing age, I begin to experience time as limited. | 🞏 | 🞏 | 🞏 | 🞏 | 🞏 | 🞏 | 🞏 |
